# An in vitro model for hepatocyte-like cell differentiation from Wharton’s jelly derived-mesenchymal stem cells by cell-base aggregates 

**Published:** 2015

**Authors:** Tahereh Talaei-Khozani, Maryam Borhani-Haghighi, Maryam Ayatollahi, Zahra Vojdani

**Affiliations:** 1 *Laboratory for stem cell research, Anatomy Department, Shiraz University of Medical sciences, Shiraz, Iran*; 2 *Tissue Engineering Lab, Tissue Engineering Department, School of Advanced Medical Science And Technology, Shiraz University of Medical sciences, Shiraz, Iran*; 3 *Transplantation research center, Shiraz University of Medical Sciences, Shiraz, Ian*

**Keywords:** Cell aggregate, Liver, Mesenchymal stem cells, Umbilical cord

## Abstract

**Aim:**

The present study investigated the differentiation potential of human Umbilical Cord Mesenchymal Stem Cells (UCMSCs) into hepatic lineage through embryonic body-like aggregate formation in the presence of IGF-1.

**Background:**

Cells derived from Wharton’s jelly have been reported to display a wide multilineage differentiation potential, showing some similarities to both embryonic (ESC) and mesenchymal stem cells (MSCs).

**Patients and methods:**

Human MSCs isolated from the umbilical cord were plated in 20 μL micro drops. A two-step differentiation protocol was used and the cell aggregates were exposed to the media supplemented with IGF, HGF, oncostatin M, and dexamethasone for 21 days. Immunoperoxidase and immuno-fluorescence were performed for cyrokeratins 18, 19 and albumin. Functional assays were done by periodic acid Schiff (PAS) and indocyanine green.

**Results:**

The expression of cytokeratin 19 was shown to be higher in the cells derived from 3D spheroids compared to those cultured in conventional protocol. They showed a polygonal shape after being exposed to hepatogenic media. Immunostaining demonstrated the expression of cytokeratin-18, 19 and albumin by the differentiated cells. Besides, PAS staining revealed glycogen storage in differentiated cells. Also, a greater number of large size differentiated cells were found at the periphery of the expanded cell aggregates.

**Conclusion:**

We established a protocol for UCMSC differentiation into hepatocytes and these cells were morphologically and functionally similar to hepatocytes. Thus, hepatocyte differentiation may be facilitated by the UCMSCs aggregate formation before administration of the differentiation protocols.

## Introduction

 Wharton’s jelly is a good source of Mesenchymal Stem Cells (MSC) that can be used without any ethical concerns. Wharton’s jelly MSC exhibits a modulatory effect on the immune response (1) therefore; it can be an appropriate cell source to be applied for cell therapy. These cells can differentiate into low immunogenic hepatocyte-like cells (2) under a proper culture condition. 

The transcriptome profile of Wharton’s jelly MSCs showed a wider range of lineage markers such as those for endoderm, besides ectoderm and mesoderm compared to the other fetal and adult MSCs (3). Wharton’s jelly-derived MSCs also express markers for both embryonic stem cell and adult MSC (4). they exhibit the potential to differentiate into all three lineages. In comparison to other MSC sources, Wharton’ jelly MSCs can generate more functional differentiated cells, such as insulin producing cells from the endoderm lineage (5). The MSCs isolated from this source are more primitive compared to the others; however, the reports implicate the lack of tumorigenicity after transplantation (6). Umbilical cord MSCs can express a low level of some hepatocyte markers, such as alpha-fetoprotein and albumin. Based on these findings, the umbilical cord derived-MSCs were considered as an appropriate source for cell based therapy of liver diseases (7).

Wharton’s jelly MSC has been demonstrated to express a high level of transcription factors involved in liver development (8). They also produce some liver progenitor (8) and early hepatic markers (9). These features make Wharton’s jelly MSCs suitable for hepatocyte differentiation compared with bone marrow or adipose-derived MSCs. 

Up to now various protocols have been applied to induce hepatocyte differentiation in human MSC derived from the bone marrow (10, 11), adipose tissue (12), menstrual blood (13), and umbilical cord (14-16). Hepatocyte Growth Factor (HGF), Fibroblast Growth Factor (FGF), and Oncostatin M are the most commonly used factors in differentiation of MSCs toward hepatocytes. These factors are sequentially added to the culture media to mimic hepatic induction and promote their maturation. 

Insulin-like Growth Factor-1 (IGF-1) is expressed by rat septum transversum and has a role in induction of the fetal liver (17). IGF-1 has been suggested as one of the most important cytokines involved in liver regeneration. IGF-1 exerts mitogenic activity on hepatocytes during liver regeneration (18). IGF-1 is produced mainly by the liver (19) and its receptor is detected on non-paranchymal liver cells (20). The combination of HGF, dexamethasone and Oncostain M with IGF-1 has been shown to induce MSCs toward a hepatogenic fate (11, 21). 

Recently, it has been shown that culturing the MSCs in aggregation modifies their complexity and physiologic status (22). For instance, the generation of 3D spheroid by hanging drop enhances the anti-inflammatory properties (22), osteogenesis ability of the human bone marrow derived-MSC (23), as well as the chondrogenic ability of human synovial derived-MSC (24). The MSC proliferation rate showed a reduction after spheroid formation; however, it was restored at the next passages (22). The transcriptome analyses of human MSCs revealed a difference in gene expression pattern after cell aggregates formation. Human bone marrow-derived MSC aggregates expressed BMP2 more than the other cytokines (24). It has been previously shown that the primitive endoderm of the embryoid body formed from Embryonic Stem Cells (ESC) produced BMP2 (25).

In comparison to the MSCs from the other sources, Wharton’s jelly MSCs has a high proliferation rate in the conventional monolayer culture systems (26). Of course, it is convenient to have a protocol to temporarily reduce the cell proliferation rate during the differentiation procedure. The researchers have to use MSCs from high passages (15) to have a low level of proliferation rate; however, the cellular senescence may occur. By forming cell aggregates, we could differentiate the younger cells as early as the first passage and reduce the proliferation rate. Besides, they also express endodermal lineage markers which may form an endoderm-like structure after cell aggregation that facilitates the hepatocyte marker expression and forms functional hepatocytes. Considering the above-mentioned points, we proposed an *in vitro* model to induce MSCs into functional hepatocytes. Regarding the important roles played by IGF-1 in liver development, the aim of this study was to find if IGF-1 could induce hepatogenesis in the MSCs derived from Wharton’s jelly. 

## Patients and Methods

***Wharton’s jelly mesenchymal stem cell isolation***

Mesenchymal stem cells were isolated from the umbilical cords of normal full-term infants delivered by cesarean section after obtaining informed consents from their parents. The umbilical cords were delivered to the laboratory in phosphate buffer saline (PBS) containing penicillin/streptomycin within 3-24 h. They were flashed by PBS and the amnion was scrapped. Then, the lumen of the vein was opened, the endothelial cells were scrapped, and the arteries were removed. The rest of the umbilical cord was cut into the pieces. Each piece was put into a 100mm culture palate dish and bathed with α-MEM containing 10% FBS, 0.1 L-glutamine and 0.1% penicillin/streptomycin. The culture media were changed every week. 

***Phenotypic analysis***

The CD markers of the expanded cells were evaluated by flow cytometry. The samples were harvested and incubated with permeabilization buffer containing tween 20 and goat serum. Then, the cells were treated with FITC- conjugated anti- CD44, CD144, PE-conjugated anti CD106, CD34, and preCP-conjugated anti CD105 antibodies (all from Abcam, UK, Cambridge). The cells were fixed with 4% paraformaldehyde and the frequencies of the positive cells were evaluated by flow cytometry. Non-specific binding was excluded by matched isotypes. A four color FACScalibur flow cytometry machine with CellQuest pro software for data acquisition was used to analyze the positive-reacted cells to various antibodies. The results were depicted as graphs using WinMed free software. 

***Osteogenic differentiation procedures***

For osteogenic differentiation, Wharton’s jelly derived-MSCs were incubated in the NH-OsteoDiff Medium (MACS, Germany) for four weeks. Then, the culture media were aspirated and the induced cells were washed and stained with 0.5% alizarin red S in PBS. 

**Table 1:** The percentage of positive cells for cytokeratins 18 and 19 cultured in conventional culture condition and 3D spherule form. The experimental cultures exposed to hepatogenic media and control cells were grown in DMEM. (n=3).

	Conventional culture condition		3D spherule formation	
	Experimental cultures	Control cultures	Experimental cultures	Control cultures
Cytokeratin 19	65 ±4	9±0.7	62.4±3	39.4±3.8
Cytokeratin 18	76±4	36±5	76.7±7	35.1±6.5

***Adipogenic differentiation procedures***

To test the adipogenic potential of Wharton’s jelly MSCs, the cells were stimulated by being cultured in DMEM supplemented with human adipogenic stimulatory supplements (StemCell Technologies Inc, Canada) for three weeks. The cells were then stained with oil red.

***3D spheroid formation***

A hanging drop cell culture procedure was used to form 3D cell aggregates. The cells at the first passage were aliquoted at densities of 1000 and 8000 cells/20µL. Then, 20µL micro drops containing the cell suspension were seeded on the inner lid of a 100mm culture dish, inverted over a petri dish, and incubated at 37°C and 5% CO_2_ for 3 days. The humidity was prepared by adding distilled water to the bottom plate. The cell spheroids were then harvested and cultured in a gelatin-coated 24 well culture plate for 3-4 days according to their expansion rate. 

***Hepatic differentiation***

The hepatogenic differentiation protocol was based on the previous finding by Ayatollahi et al. (11). A two-step procedure was performed to induce the cells toward hepatogenic lineage. At the first step, the expanded 3D spheroids were exposed to DMEM containing 6%FCS, 20ng/mL IGF-I, 30 ng/mL HGF (both from Peprotech, London, UK) and 1.5 ×10^-6^ M/L dexamethasone (Sigma) for 1 week. On the second step, the culture media were also supplemented with 10ng/mL oncostatin M (OSM) (peprotech, London, UK) up to 23 days. The culture media were changed twice a week. Similar to the experimental cultures, some expanded 3D spheroids were also exposed to the media without growth factor supplementation as control cultures.

***Immunohistochemistry***

The cultures were fixed with paraformaldehyde and incubated with permeabilization buffer containing 10% goat serum, 5% Bovine Serum albumin (BSA), and 1% tween 20 in Phosphate Buffer Saline (PBS) for 20 min. Then, the cells were incubated in FITC-conjugated anti-albumin antibody (DAKO, Denmark) at a dilution of 1:30 for 1 h at room temperature. The cultures were then washed and counterstained with 4', 6-diamidino-2-phenylindole (DAPI). 

The cultures were also stained for cytokeratins 18 and 19. The cells were fixed with paraformaldehyde for 20 min. Endogenous peroxidase was blocked by 0.3% H2O2 in distilled water for 20 min in darkness. Afterwards, non-specific binding sites were blocked by PBS containing 10% goat serum for 20 min, and then the cells were incubated in mouse anti- human cytokeratins 18 (ready to use) and 19 (at a dilution of 1:20) (both from DAKO) for 45 min. After washing the samples with PBS, they were treated with super-enhancer reagent (HK518-50K, DAKO, BioGenex, Fremont, CA, USA) for 15 min at room temperature. The cells were then incubated with dextran polymer-horseradish peroxidase (HRP) (BioGenex) for 30 min at room temperature. The chromogene was 3, 3-diamino-benzidine (DAB) (DAKO, Denmark). The cells were counterstained with hematoxylin for 3-5 min. 

***Periodic Acid–Schiff Staining (PAS)***

The functional assay of the hepatocyte-like cells was performed by PAS staining for detection of the glycogen storage. The experimental and control cultures were fixed with 4% paraformaldehyde. The samples were then washed in distilled water and incubated in 0.5 % periodic acid for 5 min. After that, the cultures were washed and bathed with Schiff’s reagent for 15 min (27). The cells were counterstained with hematoxylin. 

**Figure 1:**
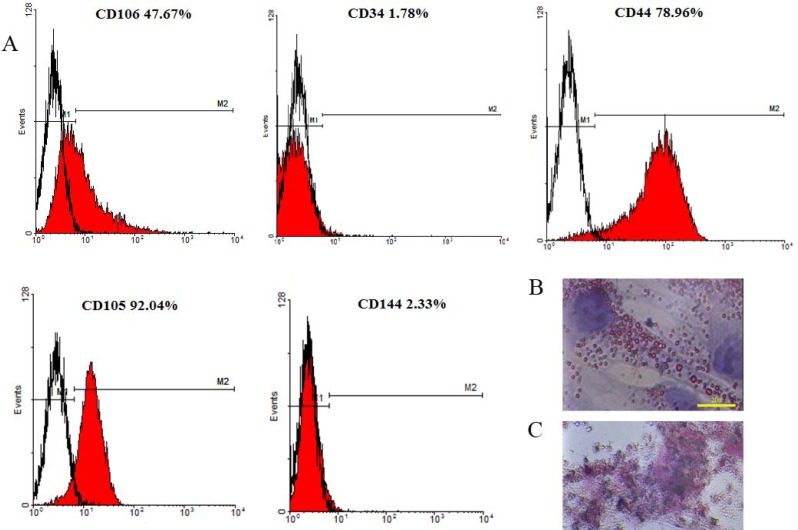
The expression of the surface markers by Wharton’s jelly-derived mesenchymal stem cells (A). The cells expressed CD44, CD105 and Cd106; however, the frequency of the positive reacted-cells for CD114 and CD34 was very low. Empty histograms indicate the staining for isotype controls while red histograms indicate the staining for specified markers. The cells also were stained with oil red and alizarin red S after treating with adipogenic (B) and osteogenic (C) media

***Indocyanine green***

To demonstrate the hepatocyte-like cell function, the indocyanine green assay was performed. The culture media were removed and the experimental and control cultures were exposed to 1 mg/mL of indocyanine green prepared in the culture media for 15 min. The micrographs were taken from the cellular uptake of indocyanine green. The staining solutions were then replaced by the fresh culture media, the cells were incubated for 6 h, and elimination of the indocyanine green was evaluated. The uptake and release of indocyanine green by HepG2 cell line was considered as a positive control. All the experiments were performed in triplicate. 

## Results

The Wharton’s jelly MSCs showed a fibroblast-like morphology. The cell size was smaller and their processes were fewer at the first passage. The cells became fusiform when reaching confluency. 

***Characterization of the Wharton’s jelly derived-mesenchymal stem cell***

The flow cytometry of Wharton’s jelly derived-MSCs revealed no reactions for hematopoietic lineage (CD34) and endothelial cell (CD144) markers. However, they showed a positive reaction for CD105, Cd106 and CD44 (Fig. 1A). Moreover, incubating the cells in osteogenic and adipogenic media showed that Wharton’s jelly-derived MSCs could differentiate into osteoblast and adipocytes, respectively (Fig. 1B and C). 

***3D spheroid formation***

**Figure 2:**
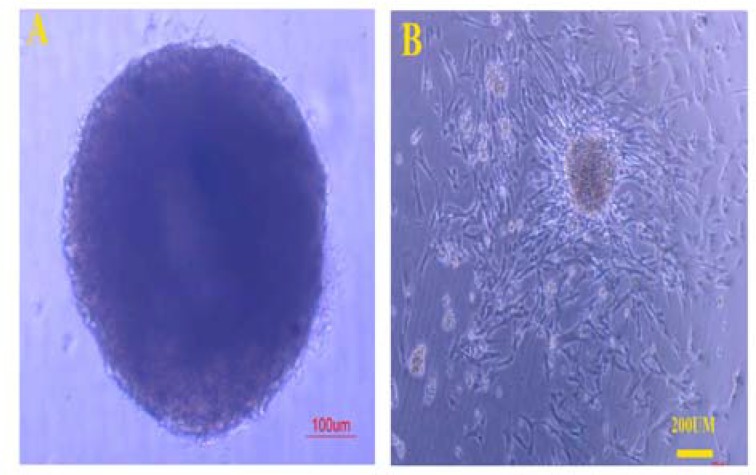
The Wharton’s jelly derived-mesenchymal stem cells formed cell aggregates in hanging drop. The cell showed similar morphology with embryoid body derived from ESCs. The 3D spheroids after culturing the cells in hanging drop for 3 days (A). The center of the cell aggregate is darker, indicating cell death. Cell expanded from the cell aggregates after culturing them in a gelatin-coated culture plate (B).

Incubating the cells in hanging drop showed that they could form cell aggregates. They were assembled as a large 3D sphere in each drop. The cells at the center of each cell aggregate showed a dark area which indicated cell degeneration (Fig 2A). When each 3D spherule was cultured in the adhesive culture dish, it started to expand (Fig 2B). The cell morphology was similar to those at the first passage; however, the proliferation rate was slower. They showed fusiform and fibroblast-like cell morphology. Furthermore, the cells close to the original cell aggregate were more compact compared to those located at the periphery. 

***Hepatic differentiation***

The cell morphology changed after being exposed to growth factors. They showed an epithelial-like and polygonal shape with very few processes. Changes in cell morphology were more obvious after adding OSM to the culture media. In the growth factor-treated cultures, the cells at the periphery of the cell colonies were larger compared to those near the center of the same cell colonies and also they were larger than the cells at the periphery of the control colonies.

**Figure 3:**
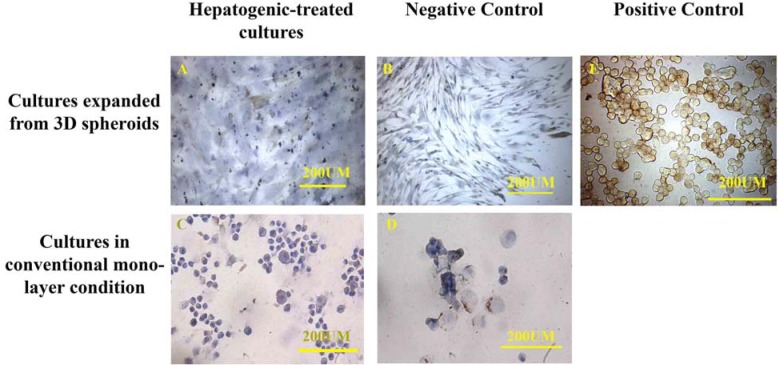
The hepatogenic-treated mesenchymal stem cells labeled by anti-cytokeratin 18 antibody showed differentiation in 3D spherules (A) and conventional monolayer culture conditions (C). Fewer control cells cultured in both 3D spherule (B) and conventional culture conditions (D) were positive for cytokeratin 18. HepG2 cell line was used as positive control (E).

**Figure 4:**
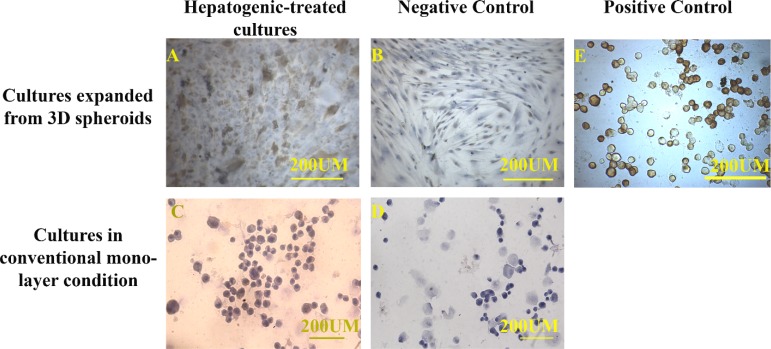
The immunocytochemistry showed hepatogenic-treated mesenchymal stem cells expressed cytokeratin 19 in both 3D spherules (A) and conventional monolayer culture (C) conditions. A higher percentage of the control cells cultured in 3D spherule condition expressed cytokeratin 19 (B) than those cultured in conventional culture condition (D). HepG2 cell line was used as positive control (E).

**Figure 5:**
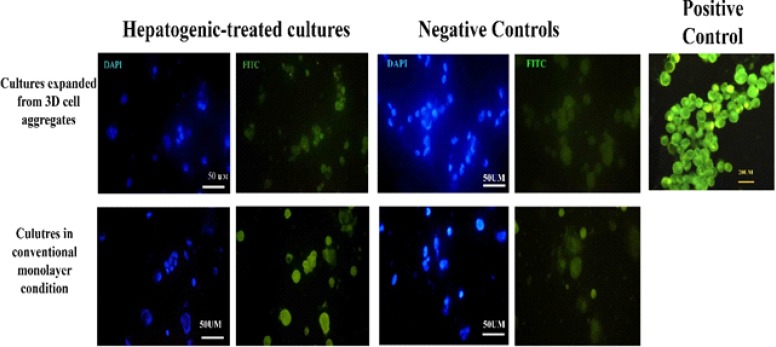
The immunofluorescence of the cells cultured in both culture conditions expressed albumin in the presence or absence of the hepatogenic media. The naive Wharton’s jelly MSCs expressed albumin. The reaction to anti-albumin antibodies was more intense in hepatogenic-treated cultures. HepG2 cell line was used as positive control.

 The immunohistochemistry of the expanded cells showed that they could express hepatocyte-specific markers such as cytokeratin 19 and 18 and albumin after 21 days. The percentages of the positive cells for cytokeratins 18 and 19 are summarized in Table 1. Although the positive cells for the hepatocyte markers were observed in both control cultures, the frequency of the positive cells for hepatocyte markers in the cultures exposed to hepatogenic media was higher than that of the control cultures. Besides, the intensity of the reaction for both cytokeratins was higher in the cells treated with hepatogenic media compared to those considered as the control cultures (Figs 3, 4). More differentiated cells were observed at the periphery of the cell expansion from spherules. The comparison of both control cultures showed that the percentage of cytokeratin 19-reacted cells was higher in the cells expanded from 3D spherules than those cultured in conventional culture condition. Both differentiated and control cells were stained with anti-albumin antibody; however, the intensity of the reaction of growth factor-treated cells for anti-albumin antibody was stronger in comparison to those in the control culture (Fig 5).

**Figure 6:**
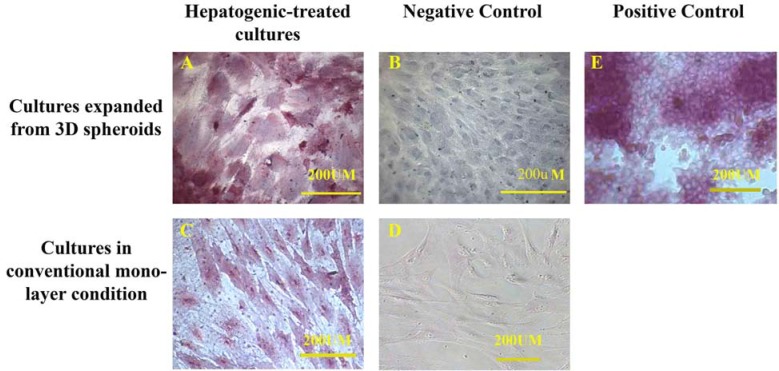
The PAS staining showed glycogen storage in the hepatogenic-treated cells cultured in both conditions, expanded from 3D spheroids (A) and conventional monolayer (C), whereas the control cultures were not PAS positive (B and D). HepG2 cell line was used as positive control (E). The positive control was also stained with PAS.

**Functional assay**

**Figure 7:**
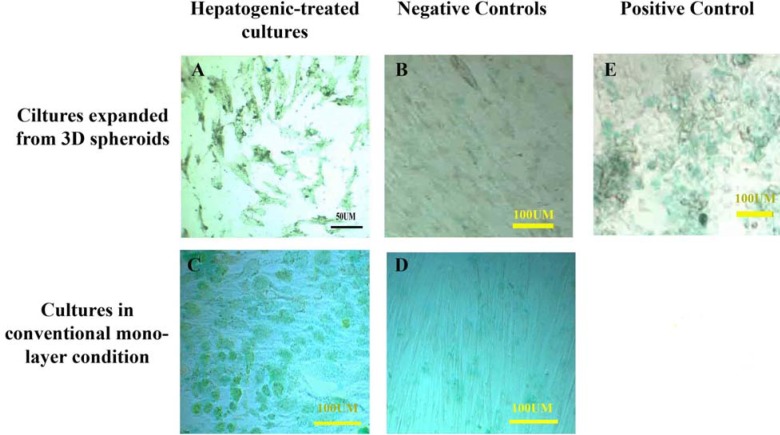
The hepatogenic-treated cells cultured in both conditions, expanded from 3D spheroids (A) and conventional monolayer culture (C), showed the uptake of indocyanine green while the non-treated mesenchymal stem cells (B and D) could not absorb it. HepG2 cell line was used as the positive control (E). The positive control also absorbed and released the indocyanine green as well.

The PAS assay showed that the cells exposed to the hepatogenic medium could store glycogen. In contrast, the non-treated cells were not PAS-positive (Fig 6). However, both cultures exposed to the hepatogenic media could store glycogen, and the reactions of the cells expanded from 3D spherules were more intense than those cultured in conventional culture condition (Fig 7). 

Also, the treated cells could uptake the indocyanine green and release it again (Fig 5). 

## Discussion

The molecular mechanism controlling the liver development and regeneration revealed that the growth factors such as HGF and IGF had pivotal roles in hepatocyte proliferation and differentiation. Hepatocyte growth factor is critical for the expression of definite endoderm markers and hepatoblast growth (28). Besides, OSM along with corticosteroids can induce hepatocyte differentiation (29). IGF-1 can stimulate ESCs to differentiate toward hepatocyte (30). The role of IGF-1 has also been shown in liver development and regeneration. IGF-1 can be released in the damaged liver, inducing the initiation of mitosis and stimulating the secretion of HGF (31,32). It has been shown that IGF-1 leads to an increase in HGF and serum albumin (33). In vitro differentiation of human bone marrow-derived MSCs has been shown by supplementation of the culture media with IGF-1 along with HGF, OSM and dexamethasone (11). The finding of the present study also confirmed that this combination of growth factors could stimulate hepatogenesis in the MSCs isolated from the other sources such as Wharton’s jelly. These findings along with the previous research (11) showed that IGF-1 has a co-stimulatory effect on cell differentiation toward hepatocyte-like cells along with HGF, OSM and dexamethasone.

Cell-cell contact in 3D spherules differs from the monolayer MSC culture condition. The cell behavior is also different in these two culture conditions. Therefore, proliferation rate and the immunoreactions of the cells can be modified by changes in the cell-cell contact pattern (22, 4). Although the results showed that the morphology of the 3D spherules was very similar to that of the embryoid body formed by ESCs, it has not been determined yet whether these cell aggregates from different sources (Wharton’s jelly and ESC) have the same histological structure and cytokine production profile. However, culturing the fetal hepatoblast at a high density has been shown to stimulate many hepatocyte metabolic functions (34). Our results showed that the Wharton’s jelly-derived MSCs could also differentiate into functional hepatocyte-like cells after cell aggregate formation.

Wharton’s jelly derived MSCs have been shown to express a low level of embryonic stem cell markers (4). It has been suggested that the cells expressing ESC markers may originate from migrating primordial germ cells. These cells may remain within the umbilical cord during the embryonic period (35). The similarities between the Wharton’s jelly-derived MSCs behavior and that of the ESC may be attributed to the origin of this subpopulation. Also, the similarity of the 3D spherules from Wharton’s jelly-derived MSCs to embryoid body from the ESC may be attributed to the origin of this subpopulation in the umbilical cord. Both embryonic stem cell and primordial germ cell lines have the same behavior, morphology and markers. Wharton’s jelly-derived MSCs could express all three lineage specific markers (3). Our study findings also indicated a low level of the endodermal marker, albumin, in the non-treated cultures. The distribution pattern of the differentiating cells showed that most of the hepatocyte-like cells derived from the MSCs were located at the periphery of the expanded colonies. Similar results have also been reported by culturing the mouse embryonic stem cells in hanging drops and then in adhesive culture plate. Albumin-positive cells were reported to appear in the surrounding cells (36). In the current study along with the previous findings, the presence of albumin was shown in the cytoplasm of naïve Wharton’s jelly MSCs. Albumin is a main constituent of serum-supplemented culture medium. Albumin has been demonstrated to be either synthesized by naive Wharton’s jelly MSCs (3, 4), or probably absorbed from the serum supplement in the culture medium. 

Combination of IGF-1, HGF, Oncostatin M and dexamethasone could induce the cells grown from a 3D cell aggregate and in conventional culture condition to express hepatocyte markers. The PAS and indocyanine green assays showed that the differentiated cells were functional. Moreover, the distribution pattern of the differentiated cells was similar to that of the cells grown from the embryoid body. Further investigations are required to determine whether this protocol can lead to induction of a functional hepatocyte and can take over all its duties after transplantation.
